# High efficient key-insulated attribute based encryption scheme without bilinear pairing operations

**DOI:** 10.1186/s40064-016-1765-9

**Published:** 2016-02-19

**Authors:** Hanshu Hong, Zhixin Sun

**Affiliations:** Key Laboratory of Broadband Wireless Communication and Sensor Network Technology, Ministry Education, Nanjing University of Posts and Telecommunications, Nanjing, China

**Keywords:** ABE, Key-insulated, Without pairings, High efficiency

## Abstract

Attribute based encryption (ABE) has been widely applied for secure data protection in various data sharing systems. However, the efficiency of existing ABE schemes is not high enough since running encrypt and decrypt algorithms need frequent bilinear pairing operations, which may occupy too much computing resources on terminal devices. What’s more, since different users may share the same attributes in the system, a single user’s private key exposure will threaten the security and confidentiality of the whole system. Therefore, to further decrease the computation cost in attribute based cryptosystem as well as provide secure protection when key exposure happens, in this paper, we firstly propose a high efficient key-insulated ABE algorithm without pairings. The key-insulated mechanism guarantees both forward security and backward security when key exposure or user revocation happens. Besides, during the running of algorithms in our scheme, users and attribute authority needn’t run any bilinear pairing operations, which will increase the efficiency to a large extent. The high efficiency and security analysis indicate that our scheme is more appropriate for secure protection in data sharing systems.

## Background

With the continuing increase of network information resources, users are confronted with urgent challenges such as how to make secure data sharing with others efficiently. To help users achieve secure and flexible data access control, Sahai and Waters (2005) proposed a new notion called attribute based encryption (ABE). In this cryptosystem, data receiver’s access privileges are described by a certain number of attributes. A data receiver can get access to the ciphertext only if the attributes he owns match with the access control policy set by the data owner. Equipped with the advantages of providing secure data protection as well as flexible access control, ABE (Goyal et al. 2006; Lewko et al. 2010; Waters 2011) has become an effective tool for secure data sharing between users.

However, the efficiency of current ABE schemes is still not high enough compared to traditional public key cryptosystem. One important factor is that encryption and decryption in ABE need frequent bilinear pairing calculations. Researchers have proved that the computational complexity of bilinear pairing is much larger than that of other operations (exponential operation, multiplication, addition) in discrete group (Chen et al. 2007; Bertoni et al. 2005). In some special network systems such as wireless sensor networks (Yu et al. 2011) or body area networks (Hu and Zhang 2013; Tan et al. 2011), the computation capacity and energy resources of terminal devices are limited, frequent bilinear pairing operations may consume too much computing resources and lead to bottleneck or node failure during the process of data sharing. Consequently, to further enrich the application scenarios of ABE, it is essential to improve the efficiency by reducing the number of pairing operations. To the best of our knowledge, the elimination of pairing operation in attribute based cryptosystem is quite new in the research literature, which has not been solved yet.

Key exposure protection is another issue remains to be tackled in attribute based cryptosystem. Although many schemes have achieved forward and backward security in terms of attribute revocation (Hur 2013; Yu et al. 2010), however, the system is still at risk when key exposure happens. If the private key owned by a non-revoked user leaks, any user can use the private key to decrypt the corresponding ciphertext since the leaked private key is still a valid one. Consequently, all the potential threat calls for frequent key refreshing in attribute based cryptosystem. When key exposure happens, effective and efficient key updating mechanism should be implemented to keep the system from potential threat.

To better guarantee the security during the process of data sharing as well as minimize the total computation cost, in the paper, we do the following research:

We propose a high efficient key-insulated ABE scheme without pairings (KI-ABE-WP). In our scheme, each user’s private key corresponds to an access structure. A user can decrypt the ciphertext only if the attributes used for encryption match with the access structure he owns. Besides, we divide the system lifetime into discrete time periods. When time period evolves, only part of the private key has to be updated and the system public parameters remain unchanged. This saves a lot of computation and transmission load when attribute revocation or key exposure happens. What’s more, during the running of algorithms in our scheme, users and attribute authority (AA) needn’t do any bilinear pairing operations, which will increase the total efficiency to a large extent compared to current ABE schemes. At last, our scheme is proved to be secure under CDH hardness assumption. The high efficiency due to the elimination of bilinear pairings makes our scheme more appropriate for secure data sharing in various network systems, especially those with limited computing capacity such as wireless sensor networks, mobile communication, etc.

The rest sections are arranged as follows:

In “Related works and preliminaries” section, we introduce the related works and essential mathematical preliminaries used to construct our scheme. The security model and concrete constructions of our scheme are proposed in “Models and assumptions” and “Constructions to our KI-ABE-WP” sections respectively. The security and performance analysis are given in “Security proof and performance analysis” section. At last, we conclude our paper and make prospects on future directions in “Conclusion” section.

## Related works and preliminaries

### Related works

Existing literatures have achieved much progress in ABE with respect to fine-grained access control (Goyal et al. 2006; Waters 2011; Bethencourt et al. 2007; Goyal et al. 2008), user flexible revocation (Hur and Noh 2011; Yu et al. 2011) and attribute based signcryption (Wang and Huang 2011), etc. Meanwhile, ABE has been widely designed for providing data protection in various network systems such as personal health record system (Li and Yu 2013), body area networks (Hu and Zhang 2013; Tan et al. 2011), wireless sensor networks (Yu et al. 2011), cloud computing (Yang et al. 2012). However, these schemes may not be entirely realistic to be applied to some application scenarios thanks to the heavy computation cost from bilinear pairing operations. Take the proposed scheme in Xhafa et al. (2015) for instance, if the number of attributes involved in encryption is *n*, then the decryption will take 4*n* times of pairing operations, which will bring a heavy computation burden on terminal devices. Consequently, to further improve the efficiency and performance of ABE, the number of pairing operations should be reduced, even totally eliminated.

Besides efficiency, key exposure protection is another issue urgently to be solved in ABE. Many existing schemes have guaranteed forward and backward security when attribute revocation happens by introducing a proxy re-encryption server (Hur and Noh 2011; Yu et al. 2010). However, these schemes only focus on the key regeneration of the revoked users, but neglect the key updating for non-revoked users. If a non-revoked user’s private key leaks, the confidentiality of the system will be threatened since the leaked private key is still a valid one. In fact, in attribute based cryptosystem, key refreshing is more important since either attribute revocation or private key exposure protection calls for frequent key-updating. Xu and Martin (2012) proposed an ABE scheme with secure key refreshing in, but their scheme has to regenerate the master key and public parameters in the system, this will bring about much more computation overheads when key updating happens. Key-insulation (Dodis et al. 2002) is a promising tool to guarantee forward and backward security as well as achieving high efficiency of key updating. In this mechanism, the lifetime of the system is divided into discrete periods. The public key remains unchanged throughout the lifetime, while temporary secret keys are updated periodically. Key-insulation mechanism can provide full security when user’s private key exposure happens and it has been designed for effective key exposure protection in identity based cryptosystem (Zhu et al. 2014), certificateless cryptosystem (Chen et al. 2015), etc. The advantage of key-insulation mechanism can also be combined into attribute based cryptosystem and propose a key-insulated ABE scheme with efficient and secure key updating.

### Hardness assumptions

- Discrete logarithm assumption (DL):

Given $X,P \in G,$ it is computational infeasible to calculate the value of *a*$(a \in Z_{q}^{*} )$ such that *X* = *aP* with a non-negligible probability within probabilistic polynomial-time.

(b) Computational Diffie–Hellman assumption (CDH):

For $a,b \in Z_{q}^{*} ,$ given (*p*, *ap*, *bp*), it is computational infeasible to calculate the value of *abp* with a non-negligible probability within probabilistic polynomial-time.

## Models and assumptions

### A real world example of KI-ABE-WP

**Fig. 1:**
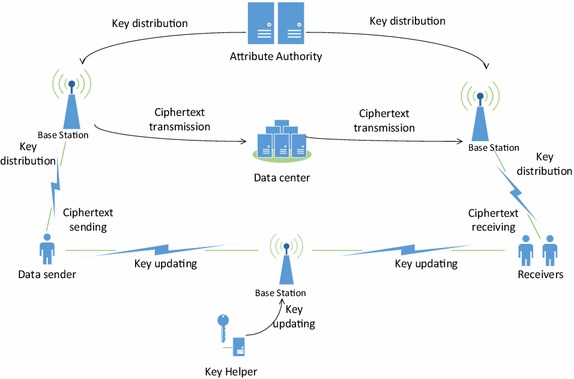
Apply KI-ABE-WP for mobile communication system

One typical application of our KI-ABE-WP is the mobile communication system, which can be illustrated in Fig. 1. It consists of six entitles: AA, key helper, base station, data centre, data sender and receiver. Base station and data centre are hardware architectures which are responsible for mobile communications and file storage. AA generates initial attribute private keys for each user in the system and the private key corresponds with an access structure. Data sender and receiver are the two sides of communication, data sender encrypts the file with a set of attributes, while a receiver can decrypt the ciphertext if the attributes used for encryption match with the access structure he owns. When system evolves into a new time period, users in the system update their private keys to the latest version with the assistance of key helper. Due to the elimination of bilinear pairing operations, the proposed KI-ABE-WP will relieve the terminal devices from heavy computation burden, thus improving the efficiency and performance of the whole system.

### Formalized definition of the algorithms in KI-ABE-WP

Our KI-ABE-WP consist of five algorithms:

- *Setup*(1^*λ*^){*PK*, *MK*} This algorithm takes a security parameter *λ* as input and outputs the public parameter *PK* and master key *MK*. *PK* is shared by users while *MK* is kept private by AA.
- $Initial\,private\,key\,generation{:}\,\{ PK,MK,\gamma ,TP_{0} \} \to \{ TD_{{\gamma ,TP_{0} }} \}$ This algorithm is operated by AA. It takes *PK*, *MK*, initial time period *TP*_0_ and the user’s access structure *γ* as input. The output of this algorithm is user’s initial private key $TD_{{\gamma ,TP_{0} }} .$
- $Key updating{:}\,\{ PK,MK,\gamma ,TP_{n} \} \to \{ TD_{{\gamma ,TP_{n} }} \}$ This algorithm is an interaction between AA and user. On input *PK*, *MK*, *γ* and the current time period *TP*_*n*_, AA outputs the key-updating component $U_{{\gamma ,TP_{n} }}$ and transfers it to users. User updates his temporal private key to the latest version using $U_{{\gamma ,TP_{n} }} .$
- *Encrypt*$\{ PK,M,\{ A_{i} \} \} \to \{ CT\}$ This algorithm is operated by the data sender. It takes *PK*, a plaintext *M* and an attribute set {*A*_*i*_} as input and outputs the corresponding ciphertext *CT*.
- $Decrypt{:}\,\{ D_{i,\gamma } ,CT\} \to \{ M\}$ This algorithm is run by the data receiver. The algorithm takes as input the ciphertext *CT* and the receiver’s temporal private key $TD_{{\gamma ,TP_{n} }} ,$ it outputs the plaintext *M*.

### Security model of KI-ABE-WP

#### **Definition**

Our KI-ABE-WP scheme is secure under chosen ciphertext attacks if there exists an *Adversary* has non-negligible advantage in the following game played by a *Challenger* and an *Adversary*.

#### Phase 1 *Setup*

*Challenger* runs *Setup* procedure to obtain the system parameters *PK* and master keys *MK*. It sends *PK* to *Adversary*.

#### Phase 2 *Queries*

*Adversary* can make the following queries to *Challenger*.

#### *Initial private key generation query*

*Challenger* can obtain user’s initial private key $D_{{\gamma ,TP_{0} }}$ by running *Initial private key generation* algorithm and returns the result back to *Adversary*.

#### *Temporal private key generation query*

*Challenger* can obtain user’s temporal private key at the current time period and returns the result $D_{{\gamma ,TP_{n} }}$ back to *Adversary*.

#### *Decrypt query*

*Adversary* can ask *Decryptquery* for ciphertext *CT*. *Challenger* runs *Decrypt* algorithm and returns the results to *Adversary*.

#### Phase 3 *Challenge*

*Adversary* chooses two plaintexts *M*_0_ and *M*_1_ and a challenging access structure $\gamma^{*}$ at current time period.

*Challenger* chooses $\sigma \in \{ 0,1\}$ randomly and calculates *CT*_*σ*_ = *Encrypt*(*PK*, *M*_*σ*_, {*A*_*i*_}) and returns the result to *Adversary*.

*Adversary* outputs a value $\sigma^{*}$ as a conjecture of *σ*.

During the whole process of the challenge game:

*Adversary* cannot ask *Challenger* for *Decryptquery* of *M*_0_ and *M*_1_.

*Adversary* cannot ask *Challenger* for *Temporal private key generation query* for the challenging structure $\gamma^{*} .$

If $\sigma^{*} = \sigma$ then *Adversary* wins the game.

We denote $Adv(A) = \left| {Pr[\sigma^{*} = \sigma ] - \frac{1}{2}} \right|$ to be the *Adversary*’*s* advantage in the above challenge game.

## Constructions to our KI-ABE-WP

### Concrete algorithms of KI-ABE-WP

#### Setup

Let *G* to be a cyclic addition group. Denote *q* and *p* to be the prime order and generator of *G* respectively. AA defines a global attribute set $$\{ A_{i} \}$$ and picks $$t_{i} \in Z_{q}^{*}$$ for each attribute in $$\{ A_{i} \}$$. Let *T*_*i*_ = *t*_*i*_*p* to be the public key of *A*_*i*_. Picks $$k_{n} \in Z_{q}^{*}$$ for each time period *TP*_*n*_ in the system lifetime. Let *K*_*n*_ = *k*_*n*_*p*. Chooses a secret number $$y \in Z_{q}^{*}$$ and calculates *Y* = *yp*. Define two hash functions $$H_{1} {:}\{ 0, 1\}^{*} \to Z_{q}^{*} ,$$$$H_{2} {:}\{ 0, 1\}^{*} \to \{ 0,1\}^{m} ,$$*m* is the size of plaintext. Define a Lagrange interpolation function $$\Delta _{i,S(x)} = \prod\nolimits_{j \in S,j \ne i} {\frac{x - j}{i - j}} .$$

The system public parameters are $\{ G,q,p,A_{i} ,T_{i} ,K_{n} , Y,H_{1} ,H_{2} \}$ and the system master keys are {*t*_*i*_, *y*, *k*_*n*_}.

#### Initial private key generation

AA randomly chooses a polynomial *q*_*x*_ for each node *x* in the user’s access tree *γ*. Denote *d*_*x*_ to be the degree of *q*_*x*_ and *thr*_*x*_ to be the threshold value node. Let $d_{x} = thr_{x} - 1.$ For the root node AA sets $q_{root} (0) = y.$ For any other node (except for root node) in the access tree, let $q_{x} (0) = q_{parent(x)}^{index(x)} .$ The initial attribute private key at time period *TP*_0_ for access structure *γ* can be denoted by $TD_{{\gamma ,TP_{0} }} = \{ q_{x} (0) + t_{i} + k_{0} \cdot H_{1} (T_{i} ,TP_{0} ),\,i \in \gamma \} .$

#### Key updating

When time period evolves from *TP*_*n*_ to *TP*_*n*+1_, AA calculates the updated key component $U_{{\gamma ,TP_{n + 1} }} = (k_{n + 1} \cdot H_{1} (T_{i} ,TP_{n + 1} ) - k_{n} \cdot H_{1} (T_{i} ,TP_{n} ),\,i \in \gamma )$ and transfers it to user. User calculates $TD_{{\gamma ,TP_{n + 1} }} = TD_{{\gamma ,TP_{n} }} + U_{{\gamma ,TP_{n + 1} }}$ as the temporal private key at time period *TP*_*n*+1_.

#### Encrypt

At time period *TP*_*n*_, for a plaintext *M*,data sender picks a random number $s \in Z_{q}^{*}$ and calculates:

1 $$\begin{aligned} & C_{1} = sp,\quad C_{2} = sT_{i} \\ & C_{3} = sK_{n} ,\quad C_{4} = H_{2} (sY) \oplus M \\ \end{aligned}$$

Then data sender sends $CT = \{ C_{1} ,C_{2} ,C_{3} ,C_{4} \}$ to data receiver.

#### Decrypt

Upon receiving *CT*, receiver calculates:

2 $$M = H_{2} \left( {\mathop \sum \limits_{i \in \gamma } \left( {TD_{{\gamma ,TP_{n} }} \cdot C_{1} - C_{2} - C_{3} \cdot H_{1} \left( {T_{i} ,TP_{n} } \right)} \right)} \right) \oplus C_{4}$$

### Correctness proof

If *x* is a leaf node, the calculation process is as follows:

3 $$\begin{aligned} DecryptNode\left( {x,TD_{{\gamma ,TP_{n} }} ,C_{1} ,C_{2} ,C_{3} } \right) & = TD_{{\gamma ,TP_{n} }} \cdot C_{1} - C_{2} - C_{3} \cdot H_{1} \left( {T_{i} ,TP_{n} } \right) \\ & = \left( {q_{x} (0) + t_{i} + k_{n} \cdot H_{1} \left( {T_{i} ,TP_{n} } \right)} \right) \cdot sp \\ & \quad - sT_{i} - sK_{n} \cdot H_{1} \left( {T_{i} ,TP_{n} } \right) = q_{x} (0) \cdot sp + t_{i} \cdot sp \\ & \quad + k_{n} \cdot H_{1} \left( {T_{i} ,TP_{0} } \right) \cdot sp - sT_{i} - sK_{n} \cdot H_{1} \left( {T_{i} ,TP_{n} } \right) \\ & = q_{x} (0) \cdot sp \\ \end{aligned}$$

All the value calculated from $DecryptNode(x,TD_{{\gamma ,TP_{n} }} ,C_{1} ,C_{2} ,C_{3} )$ will be stored as *F*_*z*_. For any *F*_*z*_ ≠ 0,the algorithm calculates *F*_*root*_ (the value of root node) using Lagrange interpolation method.

If *x* is a non-leaf node, *z* is the child node of *x*, then the algorithm calculates the value of $DecryptNode(x,TD_{{\gamma ,TP_{n} }} , C_{1} ,C_{2} ,C_{3} )$ as follows:

Let *i* = *index*(*z*), $$S_{{x^{\prime } }} = \{ index(z){:}z \in S_{x} \}$$

4 $$\begin{aligned} F_{x} & = \mathop \sum \limits_{{z \in S_{x} }} F_{z}^{{\Delta_{{i,S_{{x^{{\prime }} (0)}} }} }} \\ & = \mathop \sum \limits_{{z \in S_{x} }} sp \cdot q_{z} (0)^{{\Delta_{{i,S_{{x^{{\prime }} (0)}} }} }} \\ & = \mathop \sum \limits_{{z \in S_{x} }} sp \cdot q_{parent} (z)^{{(index(z))^{{\Delta_{{i,S_{{x^{{\prime }} (0)}} }} }} }} \\ & = \mathop \sum \limits_{{z \in S_{x} }} sp \cdot q_{z} (x)^{{\Delta_{{i,S_{{x^{\prime } (0)}} }} }} \\ & = q_{x} (0) \cdot sp \\ \end{aligned}$$

Since the value of $DecryptNode(x,TD_{{i,TP_{n} }} , C_{1} ,C_{2} ,C_{3} ) = q_{x} (0) \cdot sp,$ whether *x* is a leaf node or non-leaf node, consequently, the value of root node *F*_*root*_ and the plaintext *M* can be calculated by:

5 $$\begin{aligned} F_{root} & = q_{root} (0) \cdot sp = syp = sY \\ M & = H_{2} (F_{root} ) \oplus C_{4} = H_{2} (sY) \oplus C_{4} \\ & = H_{2} (sY)H_{2} (sY)M = M \\ \end{aligned}$$

## Security proof and performance analysis

### Security proof

#### **Theorem**

*If the proposed KI*-*ABE*-*WP can be broken by an Adversary in the random oracle model, then a Simulator can be constructed to break the CDH hardness assumption in group G successfully with a non*-*negligible advantage.*

#### *Proof*

In the challenge game, if there exists an *Adversary* can break our KI-ABE-WP with an advantage (*t*, *ɛ*) in the random oracle model, then there exists a *Simulator* which can break the CDH assumption with an advantage of $\varepsilon^{\prime }$ which satisfies:

6 $$\begin{aligned} & t^{\prime } \le t + \left( {n\left( {q_{p} + q_{i} + q_{t} + 2q_{d} + 3} \right) + 4} \right) \cdot t_{sm} + n\left( {2q_{i} + 2q_{t} + 2q_{d} + 2} \right) \cdot t_{a} \\ & \quad \varepsilon^{\prime } \ge \frac{\varepsilon }{{e\left( {q_{d} + 1} \right)}} \cdot \left( {\frac{{q_{i} \cdot q_{{H_{1} }} }}{{2^{l} }}} \right)^{{q_{t} }} \cdot \left( {\frac{{q_{p} }}{{2^{l} }}} \right)^{{1 + q_{i} }} \\ \end{aligned}$$

In the lemma (6), $q_{p} ,q_{{H_{1} }} ,q_{i} ,q_{t} , q_{d}$ are the maximum numbers of *Public key generation query,H*_1_*query*, *Initial private key generation query*, *Temporal private key generation query* and *Decryptquery* respectively. Denote *t*_*sm*_ and *t*_*a*_ to be the time consumption for running a scalar multiplication operation and an addition operation respectively.

The process of the challenge game is as follows:

Phase 1 *Setup*:

*Challenger* sets the parameters as follows:

Defines a global attribute set {*A*_*i*_}. Let *G* be a cyclic addition group with prime order *q*. The generator of group *G* is denoted by *p*. Defines two hash functions: $$H_{1} {:}\{ 0,1\}^{*} \to Z_{q}^{*} ,$$$$H_{2} {:}\{ 0,1\}^{ *} \to \{ 0,1\}^{m} ,$$*m* is the size of plaintext. Randomly picks $$a,b \in Z_{q}^{*} ,$$ sets *X* = *bp*, *Y* = *ap*.

The aim of *Simulator* is to calculate the value of *abp* according to the process of the challenge game. *Simulator* plays the role of *Challenger* and runs *Adversary* as a sub-program.

Phase 2 *Queries*:

The proof skills used in our scheme resembles the method which has been proposed in Coron (2000). Without loss of generality, supposing that *Adversary* will make *Public key generation query* for an attribute *A*_*i*_ before making *Initial private key generation query*, *Temporal private key generation query* and *Decrypt query* to *Simulator*.

Then *Adversary* makes the following queries to *Simulator*:

*Public key generation query*: *Simulator* maintains a list *L*_*p*_{*A*_*i*_, *γ*, *c*, *t*_*i*_, *T*_*i*_}. When *Adversary* asks a *Public key generation query* for *A*_*i*_ in the *γ*. *Simulator* responds as follows:

Checks if *A*_*i*_ has already existed in the list *L*_*p*_. If so, *Simulator* returns the result of *T*_*i*_ to *Adversary*. If not, *Simulator* picks a biased coin $c \in \{ 0,1\}^{l}$ at random and sets $Pr[c = 0] = \theta$ while $Pr[c = 1] = 1 - \theta$. When *c* = 0, *Simulator* chooses $t_{i} \in Z_{q}^{*}$ and sets *T*_*i*_ = *t*_*i*_*p*. Otherwise let *T*_*i*_ = *t*_*i*_*X*. *Simulator* adds the tuple {*A*_*i*_, *γ*, *c*, *t*_*i*_*, T*_*i*_} into *L*_*p*_ and sends *T*_*i*_ to *Adversary*.

*H*_1_*query*: *Simulator* maintains a list $L_{{H_{1} }} \{ A_{i} ,\gamma ,T_{i} ,TP_{n} ,H_{1} (T_{i} ,TP_{n} )\}$. When *Adversary* asks a *H*_1_*query* for *A*_*i*_, *Simulator* responds as follows:

Checks if *A*_*i*_ has already existed in $L_{{H_{1} }}$. If so, *Simulator* sends the result back to *Adversary*. If not, *Simulator* calculates the value of *H*_1_(*T*_*i*_, *TP*_*n*_) and adds it into the $L_{{H_{1} }} .$

*Initial private key generationquery*: *Simulator* maintains a list $L_{i} \{ A_{i} ,\gamma ,TD_{{\gamma ,TP_{0} }} \} .$ When *Adversary* asks a *Initial private key generationquery* for *γ*, *Simulator* responds as follows:

Checks if $\gamma$ exists in the list *L*_*p*_{*A*_*i*_, *γ*, *c*, *t*_*i*_, *T*_*i*_}. If not, *Simulator* aborts the challenge game and outputs failure. We denote this incident by *E*_1_.

Otherwise, *Simulator* randomly chooses a polynomial *q*_*x*_ for each node *x* in the user’s access tree *γ*. Denote *d*_*x*_ to be the degree of *q*_*x*_ and *thr*_*x*_ to be the threshold value node. Let $d_{x} = thr_{x} - 1.$ For any other node (except for root node) in the access tree, let $q_{x} (0) = q_{parent(x)}^{index(x)} .$*Simulator* chooses $k_{0} \in Z_{q}^{*}$ and sets initial private key $TD_{{\gamma ,TP_{0} }} = \{ q_{x} (0) + t_{i} + k_{0} \cdot H_{1} (T_{i} ,TP_{0} ),\,i \in \gamma \}$. Then simulator adds the tuple into *L*_*i*_ and sends $TD_{{\gamma ,TP_{0} }}$ to *Adversary*.

$$Temporal private key generation query{:} Simulator$$ maintains a list $$L_{t} \left\{ {A_{i} ,\gamma ,TP_{n} ,TD_{{\gamma ,TP_{n} }} } \right\}$$. When *Adversary* asks a *Temporal private key generation query* for *γ*, *Simulator* responds as follows:

Checks *γ* in the list $L_{i} \{ A_{i} ,\gamma ,TD_{{\gamma ,TP_{0} }} \} .$ If *γ* does not exist in *L*_*i*_, *Simulator* aborts the challenge game and outputs failure. We denote this incident by *E*_2_.

Checks $L_{{H_{1} }} \{ A_{i} ,\gamma ,T_{i} ,TP_{n} ,H_{1} (T_{i} ,TP_{n} )\} .$. If the tuple {*γ*, *A*_*i*_} does not exist in $L_{{H_{1} }}$, *Simulator* aborts the challenge game and outputs failure. We denote this incident by *E*_3_.

Otherwise, *Simulator* randomly chooses $k_{n} \in Z_{q}^{*}$ and calculates $TD_{{\gamma ,TP_{n} }} = TD_{{\gamma ,TP_{0} }} + (k_{n} H_{1} (T_{i} ,TP_{n} ) - k_{0} \cdot H_{1} (T_{i} ,TP_{0} ))$. *Simulator* sends $TD_{{\gamma ,TP_{n} }}$ to *Adversary* and adds the tuple into $L_{t} \{ A_{i} ,\gamma ,TP_{n} ,TD_{{\gamma ,TP_{n} }} \} .$

*Decrypt query*: *Simulator* maintains a list *L*_*D*_{*A*_*i*_, *γ*, *CT* = {*C*_1_, *C*_2_, *C*_3_, *C*_4_}, *M*}. When *Adversary* asks a *Decrypt query* for {*A*_*i*_, *γ*, *CT* = {*C*_1_, *C*_2_, *C*_3_, *C*_4_}}, *Simulator* responds as follows:

Checks {*A*_*i*_, *γ*} in the list *L*_*p*_{*A*_*i*_, *γ*, *c*, *t*_*i*_, *T*_*i*_}. If *c* = 1, *Simulator* aborts the game and outputs failure. We denote this incident by *E*_4_.

Otherwise, *Simulator* recovers $TD_{{\gamma ,TP_{n} }}$ from $L_{t} \{ A_{i} ,\gamma ,TP_{n} ,TD_{{\gamma ,TP_{n} }} \}$ and calculates $M = C_{4} \oplus H_{2} \left( {\sum\nolimits_{i \in \gamma } {TD_{{\gamma ,TP_{n} }} \cdot C_{1} - C_{2} - C_{3} \cdot H_{1} (T_{i} ,TP_{n} )} } \right)$. *Simulator* sends the result to *Adversary* and adds the tuple into *L*_*D*_{*A*_*i*_, *γ*, *CT* = {*C*_1_, *C*_2_, *C*_3_, *C*_4_}, *M*}.

Phase 3 *Challenge*:

*Adversary* outputs two plaintext *M*_0_ and *M*_1_ with a challenging access structure γ* at the current time period *TP*_*n*_.

*Simulator* checks if γ* exists in the list *L*_*p*_{*A*_*i*_, *γ*, *c*, *t*_*i*_, *T*_*i*_}. If not, *Simulator* aborts the challenge game and outputs failure. We denote this incident by *E*_5_. If γ* exists in the list *L*_*p*_{*A*_*i*_, *γ*, *c*, *t*_*i*_, *T*_*i*_} and *c* = 0, *Simulator* aborts the challenge game and outputs failure. We denote this incident by *E*_6_.

*Simulator* runs Temporal private key generation query for γ* and calculates $TD_{{\gamma^{*} ,TP_{n} }} = (q_{x} (0) + t_{i} + k_{n} \cdot H_{1} (T_{i} ,TP_{n} ))^{*}$. Then, *Simulator* picks $\sigma \in \{ 0, 1\}$, $s \in Z_{q}^{*}$ and calculates:

7 $$\begin{aligned} & C_{1,\sigma } = s \cdot X,\quad C_{2,\sigma } = s \cdot t_{i} X \\ & C_{3,\sigma } = s \cdot k_{n} X \\ & C_{4,\sigma } = H_{2} \left( {\mathop \sum \limits_{i \in \gamma } \left( {TD_{{\gamma^{*} ,TP_{n} }} \cdot C_{1,\sigma } - C_{2,\sigma } - C_{3,\sigma } \cdot H_{1} \left( {T_{i} ,TP_{n} } \right)} \right)} \right) \oplus M_{\sigma } \\ \end{aligned}$$

*Simulator* sends *CT*_*σ*_ = {*C*_1,*σ*_, *C*_2,*σ*_, *C*_3,*σ*_, *C*_4,*σ*_} to *Adversary*.

*Adversary* outputs $M_{\sigma }^{ *}$ as a guess of $M_{\sigma } .$ If $M_{\sigma }^{*} = M_{\sigma }$ and *Adversary* wins the game, *Simulator* outputs $abp = s^{ - 1} \cdot \sum\nolimits_{i \in \gamma } {\left( {TD_{{\gamma^{*} ,TP_{n} }} \cdot C_{1,\sigma } - C_{2,\sigma } - C_{3,\sigma } \cdot H_{1} (T_{i} ,TP_{n} )} \right)}$ as the solution to CDH assumption in group *G*.

Then we will analyse the time complexity of *Simulator* in breaking CDH assumption in group *G*.

From the description, assuming the average number of attributes involved is “*n*”, for each request of *Public key generation query*, *Initial Private key generation query*, *Temporal private keygenerationquery* and *Decryptquery*, *Simulator* has to run *n* times of multiplication operation, *n* times of multiplication operation and 2*n* times of addition operation, *n* times of multiplication operation and 2*n* times of addition operation, 2*n* times of multiplication operation and 2*n* times of addition operation respectively.

During the *Challenge* phase, *Simulator* has to run (3*n* + 4) times of multiplication operation and 2*n* times of addition operation.

Denote *t*_*sm*_, *t*_*a*_ to be the time consumption of scalar multiplication operation and addition operation in group *G* respectively. From what has been discussed above, the total time complexity of *Simulatort*′ satisfies:

8 $$t^{{\prime }} \le t + \left( {n\left( {q_{p} + q_{i} + q_{t} + 2q_{d} + 3} \right) + 4} \right) \cdot t_{sm} + n\left( {2q_{i} + 2q_{t} + 2q_{d} + 2} \right) \cdot t_{a}$$

Next we will discuss the advantage of *Simulator* in breaking the CDH assumption.

During the process of the challenge game, the responses of *Initial Private key generation query*, *Temporal private keygenerationquery* and *Decryptquery* return to *Adversary* are valid and indistinguishable if *E*1, *E*2, *E*3, *E*4 do not happen. Furthermore, if *Adversary* succeeds in distinguishing $M_{\sigma }$ and *E*5, *E*6 do not happen, then *Simulator* is capable of breaking the CDH assumption.

Next we will calculate the probability of the incidents discussed above.

According to the process of queries phase, the probability of *E*4 and *E*6 not occurring can be denoted by lemma (9):

9 $$Pr\left| {\overline{E4} \cap \overline{E6} } \right| = \theta^{{q_{d} }} \cdot (1 - \theta )$$

The value of $Pr\left| {\overline{E4} \cap \overline{E6} } \right|$ is maximized in lemma (10) when $\theta = \frac{{q_{d} }}{{1 + q_{d} }}.$

10 $$Pr_{max} \left| {\overline{E4} \cap \overline{E6} } \right| = \frac{{e^{ - 1} }}{{1 + q_{d} }}$$

Since the responses of *Public key generation query* act as random oracle model, consequently, the probability of *E*1 and *E*5 not occurring can be denoted by lemma (11):

11 $$Pr\left| {\overline{E1} \cap \overline{E5} } \right| = \left( {\frac{{q_{p} }}{{2^{l} }}} \right)^{{1 + q_{i} }}$$

Likewisely, the probability of *E*2 and *E*3 not occurring can be denoted by lemma (12):

12 $$Pr\left| {\overline{E1} \cap \overline{E5} } \right| = \left( {\frac{{q_{i} \cdot q_{{H_{1} }} }}{{2^{l} }}} \right)^{{q_{t} }}$$

Taking all the probabilities of the above incidents into account, it can be figured out that if *Adversary* successfully attacks our scheme with an advantage *ɛ*, then a *Simulator* can break the CDH assumption in group *G* with an advantage of *ɛ*′ which satisfies:

13 $$\varepsilon^{{\prime }} \ge \frac{\varepsilon }{{e\left( {q_{d} + 1} \right)}} \cdot \left( {\frac{{q_{i} \cdot q_{{H_{1} }} }}{{2^{l} }}} \right)^{{q_{t} }} \cdot \left( {\frac{{q_{p} }}{{2^{l} }}} \right)^{{1 + q_{i} }}$$

### Secure and efficient key updating

Our scheme achieves both secure and efficient key updating. According to the *Keyupdating* algorithm, the updated key component for attribute *A*_*i*_ at time period *TP*_*n*+1_ is calculated as $U_{{\gamma ,TP_{n + 1} }} = (k_{n + 1} \cdot H_{1} (T_{i} ,TP_{n + 1} ) - k_{n} \cdot H_{1} (T_{i} ,TP_{n} ),\,i \in \gamma ).$ Since a user cannot obtain the value of *k*_*n*+1_, *k*_*n*_, it is computational infeasible for him to calculate the value of $U_{{\gamma ,TP_{n + 1} }}$ and update his private keys. Without loss of generality, when a user’s private key $TD_{{\gamma ,TP_{n} }}$ was leaked during the time period *TP*_*n*_, the system still maintains safe after *TP*_*n*_ since all the private keys have been securely updated.

With respect to the computation cost, key updating for a single attribute at a discrete period only needs one multiplication operation, one addition operation and 1 *H*_1_ operation. Besides, the system parameters remain unchanged throughout different time periods and this will reduce more the communication overheads.

### Performance analysis

In this paper, assuming that the number of attributes involved in encryption is *n*, according to the algorithms discussed above, the *Encrypt* algorithm will take (*n* + 3) times of multiplication operations, one addition operation and one *H*_2_ operation, while the *Decrypt* algorithm will take *2n* times of multiplication operations, 2*n* times of addition operations and 1 *H*_2_ operation. Detailed computation costs of each algorithm is listed in Table 1.

**Table 1:** Computation cost of algorithms in our scheme

Algorithms	Setup	Initial private key generation	Encrypt	Decrypt	Key updating
Multiplication	n + 1	n	n + 3	2n	n
Addition	0	2n	1	2n	n
Hash 1	0	n	0	0	n
Hash 2	0	0	1	1	0

From Table 1, it can be seen that the total computation efficiency is much higher in our scheme compared to current ABE schemes since bilinear pairing operations have been totally eliminated.

## Conclusion

In this paper, we combine the advantage of key-insulation mechanism with ABE and propose a high efficient key-insulated ABE algorithm without pairings (KI-ABE-WP). During the running of algorithms in our scheme, users and AA needn’t run any bilinear pairing operations. The high efficiency and proved security make our scheme more appropriate for data sharing in network systems, especially those with limited computing capacity such as wireless sensor networks, mobile communication system, etc.

Our future research should focus on the ABS (attribute based signature) without pairing operations, which provides secure data authentication with higher efficiency than current ABS schemes.
